# Together Apart: Evaluating Lichen-Phorophyte Specificity in the Canarian Laurel Forest

**DOI:** 10.3390/jof8101031

**Published:** 2022-09-29

**Authors:** Cristina González-Montelongo, Israel Pérez-Vargas

**Affiliations:** Department of Botany, Ecology and Plant Physiology, Faculty of Pharmacy, University of La Laguna, 38200 La Laguna, Spain

**Keywords:** Canary Islands, epiphytic lichen, laurel forest, Macaronesia, phorophyte

## Abstract

The effects of host tree identity on epiphyte lichen communities are a controversial issue, as the results obtained in different forest environments studied are not consistent. We investigated the host preferences for lichens in the laurel forest of Macaronesia. For this purpose, we analyzed the lichen communities growing on the four most common trees (Erica canariensis Rivas-Mart., M. Osorio and Wildpret, Morella faya (Aiton) Wilbur, Laurus novoca-nariensis Rivas-Mart., Lousa, Fern. Prieto, E. Días, J.C. Costa and C. Aguiar, and Ilex canariensis Poir. in Lamarck) in the laurel forest of the Canary Islands. The diversity, richness, and lichen composition showed a repetitive and common pattern with the functional traits studied. Although the existence of specificity with respect to the phorophyte species was not demonstrated, there was a clear affinity of the epiphytic lichens to the physico-chemical features of the bark (texture and pH), canopy architecture, foliar characteristics, etc. Our results highlight the importance of the natural diversity of tree species in the laurel forest. Due to the diversity and uniqueness of the lichen species that support each of the phorophytes, this fact should be taken into account in landscape protection and restoration actions, especially in those islands where the forest is highly fragmented.

## 1. Introduction

The Macaronesian laurel forests, also called laurisilva and monteverde, are humid to hyper humid evergreen forests of the cloud belt of the Macaronesian islands. They are a value not only in terms of biodiversity but also biogeography [1]. Tree species with laurel-shaped leaves are predominant, forming a dense canopy up to 40 m high with scant vegetation in the understory [2]. Laurel forest is cataloged as a priority habitat by the European Union [3], and it is an example of a biodiversity hotspot threatened by human impact. Mankind has been altering the native forests in Macaronesia for at least five centuries. In the Canary Islands, these disturbance activities began with the aboriginal inhabitants; although they did not have sophisticated tools, they certainly used fire to create farmland and introduced livestock (sheep, goats, and other animals), as well as other plants [4,5]. After the Hispanic colonization in the 15th century, the natural landscape became increasingly transformed. In the second half of the 20th century, a significant part of this native laurel forest was cleared in the Canaries for livestock, agriculture, and planting exotic species. Due to this extensive exploitation, only 11.8% of the original laurel forest remains in this archipelago nowadays [5].

Cloud forests are considered one of the most vulnerable terrestrial ecosystems to climate change [6]. Global climate models predict a future downward shift of the cloud base in the Macaronesian region [7]. Therefore, climate change would presumably expose the laurel forests to higher radiation and temperatures and lower rainfall [8]. These changes in climatic conditions could produce an extensive change in a plurispecific forest, such as Monteverde, which has more than 20 different species in its tree stratum. Among these species, some are adapted to more arid conditions, while others survive only in the most humid and shady areas. Furthermore, these changes might affect not only the vascular flora of this forest but also the epiphytic community that depends on high atmospheric moisture conditions and the tree species on which they grow. Lichens contribute significantly to this epiphytic community, being a key component of forest biodiversity [9]. They are poikilohydric organisms, and consequently, their water content is directly regulated by environmental humidity. Physiological activity and hence growth is restricted to the hydration period, and consequently, desiccation will affect the gross metabolism, including essential processes, such as photosynthesis and respiration [10].

Lichens are one of the most remarkable organisms in monitoring and indicating environmental quality, especially atmospheric pollution [11,12], but they are also used as indicators of ecological features, such as forest conservation [13,14,15]. In addition, they provide information about microclimatic conditions, such as humidity [16,17], fog [18,19,20], solar radiations [21,22], substrate pH [23,24], etc.

Although some fungi have a broad host range and are thus not tree specific [25], things look rather different in lichen-forming fungi. The influence of host tree species on the distribution of epiphytic lichens has been studied from boreal to tropics with varied results [26,27,28,29,30,31].

Earlier studies in Macaronesian cloud forests have shown that the epiphyte–host relationships vary depending on tree identity in some groups of epiphytes, such as bryophytes [32,33,34,35]. However, even though the host tree traits may have important effects on the species diversity of epiphytic lichens, there are no studies on this subject in Macaronesia. The main aim of this study was to analyze the effect of tree species composition and host tree traits on lichen composition and richness of epiphytic lichen communities in the laurel forest of the Canary Islands. 

The present research contributes to a better understanding of this highly threatened ecosystem, where basic ecological knowledge is needed for monitoring its evolution, impacts, and even for the control and improvement of conservation measures.

## 2. Materials and Methods

### 2.1. Study Areas

This study was carried out in the Canary Islands. The main areas where the laurel forests are growing and are more abundant are located in Tenerife, La Gomera, and La Palma, so we restricted the fieldwork to these islands. The other islands do not have a proper representation of this forest because: (i) the island does not have the environmental conditions required for its presence, or (ii) logging over the last five centuries has eliminated this endemic forest [36,37,38].

**Figure 1:**
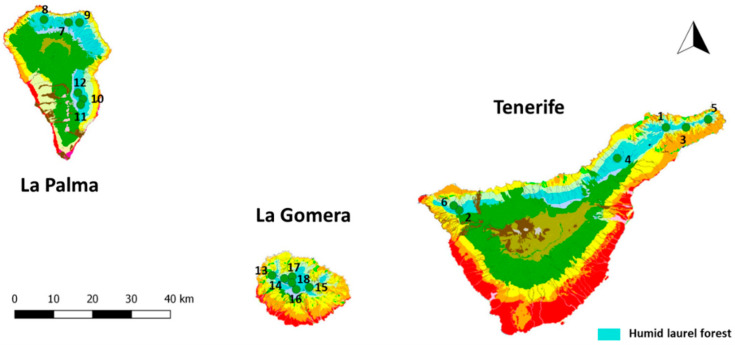
Field plots installed on potential area of humid laurel forest (*Lauro novocanariensis—Perseetum indicae*) in La Palma, La Gomera, and Tenerife islands.

Macaronesian cloud forests comprise an environmentally complex system in which different plant associations of evergreen laurel forest can be distinguished. While macroclimatic conditions in all these stands are quite similar, there are small differences in some abiotic variables, such as the altitude, exposure, topography, fog regimes, etc., so microclimatic conditions may be different and could affect the lichen communities. To minimize the effect of these differences and to homogenize our plots as much as possible, all of them were always located in the potential habitat of the humid evergreen laurel forest (Lauro novocanariensis—Perseetum indicae Oberdorfer ex Rivas-Martínez, Arnáiz, Barreno and A. Crespo 1977 corr. Rivas-Martínez et al., 2002), the most widespread association in the archipelago following Ref. [5]. (Figure 1). The best examples of this forest are found in the north of Tenerife (N), E of La Palma, and on La Gomera [39]. The fieldwork was carried out on these islands between 750 and 1100 m a.s.l. where the humid laurel forest grows (Table 1). 

The temperatures in these areas are mild throughout the year, with no frost or snow in winter. The mean annual temperature varies from 13 to 18 °C. Annual rainfall ranges from 500 to 1200 mm with a dry period in summer [40]. However, the study area is affected by the prevailing NE trade winds, which are responsible for the frequent low-level cloud cover and the resultant additional water supply deposited by fog that occurs mainly in the summer and early autumn [41].

### 2.2. Plot Selection and Sampling

Our field campaigns were authorized by Área de Medioambiente-Cabildo Insular de Tenerife, by Área de Medioambiente-Cabildo Insular de La Palma, and by Parque Nacional de Garajonay, with permission to have access and collect data (including biological material).

**Figure 2:**
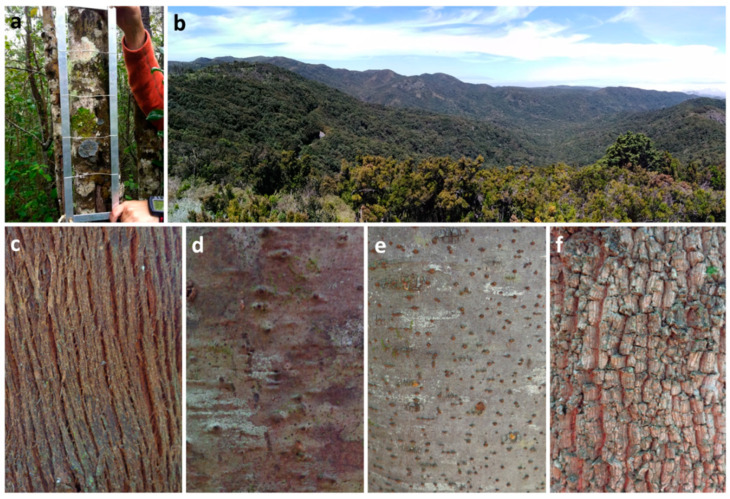
(**a**): Sampling methodology: frame on laurel forest’s tree. (**b**): Laurel forest in La Gomera Island. (**c**–**f**): Tree bark of *Erica canariensis* (**c**), *Ilex canariensis* (**d**), *Laurus novocanariensis* (**e**), and *Morella faya* (**f**).

Six sampling plots of 10 × 10 m were chosen on each island in well-preserved areas without signs of anthropogenic disturbances. In each plot, we selected two trees of *Morella faya*, two of *Erica canariensis*, two of *Laurus novocanariensis*, and two of *Ilex canariensis* (Figure 2). The laurel forest is a plurispecific forest, with about twenty tree species. Studies on host tree specificity in natural forests, where the scarcity of individuals of a given group for the many coexisting species prevents adequate replication, have often yielded inconclusive results in some groups of epiphytes [42]. Host tree species can support specific subsets of the local epiphytic species pool according to the specific properties of the trees [43,44,45]. The four selected trees were chosen not only because of their different bark properties, leaf types, architecture, and canopy but also because they are very abundant and common enough in the laurel forest to justify their presence in each selected plot.

Dead and leaning trees were avoided, and only those that had a diameter at breast height (DBH) > 22 cm were sampled. To study lichen diversity, we used a frame of 50 × 10 cm subdivided into 5 quadrats of 10 × 10 cm. The uppermost edge of the frame was located at 1.5 m above ground level, adjusted up to a maximum of 2 m if the trunk was unsuitable at the desired height, following Refs. [46,47] with some modifications in order to adjust these guidelines to the island territory (see Ref. [48]). Thus, the number of quadrats studied by tree was 10 (5 in the north orientation and 5 in the south), and 80 quadrats were studied in each sampling plot. The survey was performed between September 2013 and March 2018.

### 2.3. Specimens Identification

At each quadrat of 10 × 10 cm, all lichens (macro- and microlichens) were collected and subsequently identified in the laboratory. All details of the identification methods were described in Ref. [48]. Voucher specimens were deposited in TFC-Lich Herbarium of the University of La Laguna. The nomenclature of the lichen species mainly follows Refs. [49,50]. Species names and authors of the taxa are outlined in Supplementary Table S1, they were omitted from the text.

### 2.4. Morphological Traits and Functional Groups

The species were classified according to the traits: (1) photobiont, (2) growth form, and (3) reproductive strategy. Additionally, the species were grouped in functional groups (4) according to their ecological requirements (tolerance to eutrophication, water requirements, solar irradiation, poleotolerance, and pH of the substrate) according to Refs. [50,51] (Table 2 and Table 3). Functional groups were considered as groups of species with a similar response to an environmental factor [51]. Following Refs. [51,52], we used the maximum value available for each ecological indicator. If no ecological data were available in the literature for a given species, the values for these ecological indicators were assigned based on expert assessments by Canarian lichenologists and our own field observations.

### 2.5. Data Analysis

The statistical procedures applied are described in depth in Ref. [48]. We used a Venn diagram to show the exclusive vs. shared species among the phorophytes Venny 2.1.0 [53]. In order to determine lichen diversity, we calculated the species richness (S) and lichen diversity value (LDV), following Refs. [46,47], and studied the significant value of the medians through the medians of the Kruskal–Wallis nonparametric test (H; *p* ≤ 0.05) and the Mann–Whitney test adjusted by the Bonferroni procedure (U; *p* ≤ 0.0167). The dependence relationship between the traits and lichen functional groups and laurel forest selected trees was studied with the chi-square test. To test if the ecological requirements of lichens (pH, solar irradiation, aridity, eutrophication, and poleotolerance) are similar among the trees, we used the community-weighted mean trait value CWM [54]. We built an abundance matrix with the plot, tree, and exposure, following Ref. [55], and computed a non-metric multidimensional scaling (NMDS) with 200 permutations and the Jaccard distance [56] to reveal the spatial order of lichen composition of the four compared trees, in two dimensions. To study the adequacy of the sample configuration in the NMDS, we analyzed the stress value [57]. Finally, to determine whether there were significant differences between the phorophytes, we ran an ANOSIM test [58], and to identify the lichen species that contributed most to the similarity (and dissimilarity) among the phorophytes, we used the SIMPER analysis [58].

The S, LDV, Kruskal–Wallis, Mann–Whitney analyses, chi-square test, and boxplot were analyzed and represented using Paleontological Statistics (PAST) v.3.12 [59]. The CWM representations and dispersion graph were compiled in Microsoft^®^ Excel. NMDS, ANOSIM, and SIMPER analyses were analyzed and represented using Community Analysis Package 3.11 (CAP; [60]).

## 3. Results

### 3.1. Lichen Diversity

A total of 165 epiphytic lichen taxa (grouped in 34 families and 62 genera) were identified within more than 4000 samples collected. A complete list of all lichen species recorded is presented in Supplementary Table S1. The five most frequent families in the laurel forest of the three studied islands were: *Parmeliaceae* (32 taxa), *Ramalinaceae* (15), *Lobariaceae* (12), *Lecanoraceae* (11), and *Pertusariaceae* (10). The five most frequent species were: *Parmotrema perlatum* (202 samples), *Chrysothrix candelaris* (142), *Leucodermia leucomela* (117), *Phlyctis agelaea* (84), and *Bacidia absistens* (73). 

**Figure 3:**
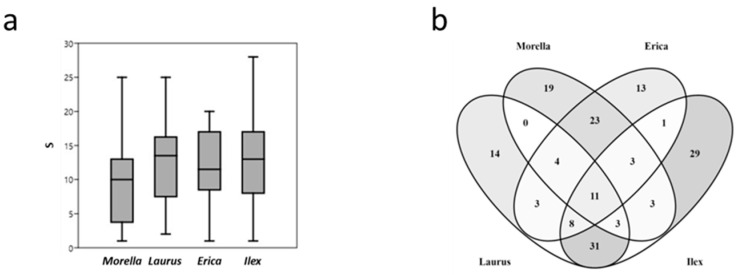
Boxplot of richness (S) by phorophyte (**a**) and Venn diagram of unique and shared lichen taxa of four phorophytes (*Erica canariensis*, *Ilex canariensis*, *Laurus novocanariensis,* and *Morella faya*) in all studied islands (**b**).

The minimum, average, and maximum number of species recorded per phorophyte were (1)—12—(20) in *Erica canariensis*, (1)—13.6—(28) in *Ilex canariensis*, (2)—12.3—(25) in *Laurus novocanariensis*, and (1)—9.5—(25) in *Morella faya*. In total, *Erica canariensis* had at least 65 taxa (in 49 genera and 18 families), *Ilex canariensis* had 89 taxa (in 53 genera and 22 families), *Laurus novocanariensis* had 74 taxa (in 41 genera and 22 families), and *Morella faya* had 66 taxa (in 32 genera and 20 families). We did not find significant differences between the phorophyte and richness (Figure 3a).

Some lichen taxa were found in all phorophytes (11, 6.7%); however, the number of species exclusive to each of the studied phorophytes was higher: 13 taxa (7.9%) in *Erica canariensis*, 14 (8.5%) in *Laurus novocanariensis*, 19 (11.5%) in *Morella faya*, and 29 (17.6%) in *Ilex canariensis*. The shared taxa between *Laurus novocanariensis* and *Ilex canariensis* (31, 18.8%), and *Erica canariensis* and *Morella faya* (23, 13.9%) stand out above the shared taxa among the other possible combinations (*Laurus novocanariensis* vs. *Morella faya*: 0 taxa; *Laurus novocanariensis* vs. *Erica canariensis*: 3; *Morella faya* vs. *Ilex canariensis*: 3; and *Erica canariensis* vs. *Ilex canariensis*: 1). In summary, there were more exclusive taxa (75) than taxa shared between two phorophytes (61), three phorophytes (18), and all phorophytes (11) (Figure 3b).

### 3.2. Traits and Lichen Functional Groups

**Figure 4:**
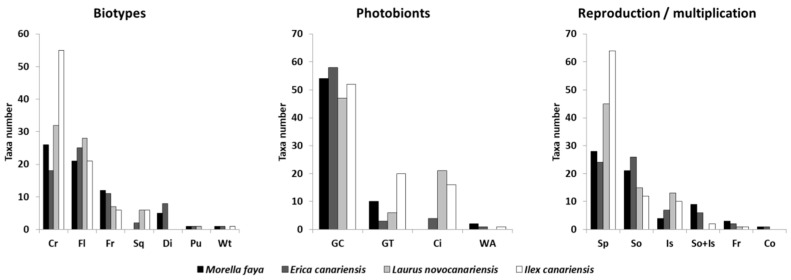
Morphological traits of lichen in *Morella faya* (black bars), *Erica canariensis* (dark gray bars), *Laurus novocanariensis* (gray bars), and *Ilex canariensis* (white bars); biotypes: Cr: crustaceous, Fl: foliose, Fr: fruticose, Sq: squamulose, Di: dimorphic, Pu: pulverulent/leprose, Wt: without thallus; photobionts: GC: green clorococcoid algae, GT: Trentepohliaceae algae, Ci: cyanobacteria, WA: without algae; and reproduction/multiplication: Sp: spore, So: soredia, Is: isidia, So + Is: soredia and isidia, Fr: fragmentation, Co: conidia.

The crustose biotype is predominant in the four studied phorophytes, followed by foliose (44.4% and 32.2%, respectively). Green algae clorococcoid photobiont predominates (71.5%), followed by green algae *Trentepohliaceae* (13.2%) (mainly in Ilex canariensis) and cyanobacteria (13.9%) (cyanolichens primarily in *Laurus novocanariensis* and *Ilex canariensis*). Epiphytic lichens in all phorophytes disperse mostly sexually (54.6%); this is more noticeable in species growing on *Laurus novocanariensis* and *Ilex canariensis* than on *Morella faya* and *Erica canariensis*. Regarding asexual strategy, dispersion by soredia (25.1%) is the most frequent propagation type, followed by isidia (11.5%) and both types combined (5.8%) (Figure 4).

**Figure 5:**
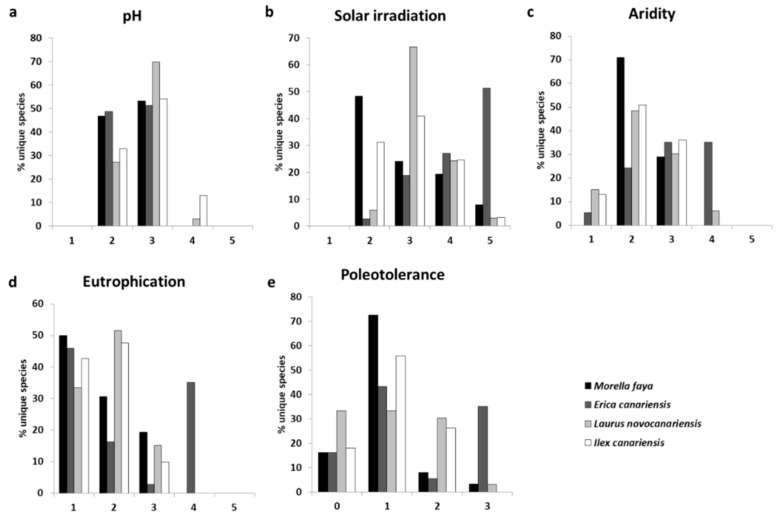
Percentage of unique species in each category of functional groups value and phorophytes studied: (**a**) substrata pH, (**b**) solar irradiation, (**c**) aridity, (**d**) eutrophication, and (**e**) poleotolerance.

Regarding the ecological conditions preferred by the recorded lichens, Figure 5 summarizes the responses of the exclusive species (or unique species, i.e., those lichens that only grow on a certain phorophyte) occurring on the studied trees concerning the pH, solar irradiance, aridity, eutrophication, and poleotolerance. The epiphytic lichens of *Morella faya* and *Erica canariensis* share pH substrate categories 2 (“*on acid substrate*”) and 3 (“*on subacid to subneutral substrate*”), whereas the epiphytic lichens of *Laurus novocanariensis* and *Ilex canariensis* are mainly associated with substrate pH level 3 (Figure 5a). The response of epiphytic lichens to solar irradiation differs among all the studied phorophytes; the lichen composition of *Morella faya* is typical of “*shaded situations*” (category 2) and “*sites with plenty of diffuse light but scarce direct solar irradiation*” (category 3), followed by “*sun-exposed sites, but avoiding extreme solar irradiation*” (category 4), but some species present in *Morella faya* are typical of “*sites with very high direct solar irradiation*” (category 5). A similar response is observed in the epiphytic lichens of *Ilex canariensis*, with most lichens in categories 2 and 3, followed by category 4. The epiphytic composition of *Laurus novocanariensis* is mainly grouped in category 3, followed by category 4. Finally, *Erica canariensis* composition differs from that of other phorophytes, with lichen species typical of illuminated sites being more abundant (category 5, followed by categories 4 and, to a lesser extent, 3) (Figure 5b). We did not observe any species typical of a “*very xerophytic*” environment (category 5). The lichen composition of *Morella faya*, *Laurus novocanariensis,* and *Ilex canariensis* is shared between categories 2 (“*intermediate between hygrophytic and mesophytic stands*”) and 3 (“*mesophytic*”), and the composition of *Erica canariensis* is shared among categories 3 and 4 (“*xerophytic, but absent from extremely arid stands*”), followed, to a lesser extent, by category 2 (Figure 5c). We did not find any species typical of “*very high eutrophication*” (category 5). The lichen composition of *Laurus novocanariensis* and *Ilex canariensis* has a similar response to eutrophication—typical of “*very weak eutrophication*” stand (category 2), followed by “*no eutrophication*” site (category 1) and “*weak eutrophication*” environment (category 3). The lichen composition of *Morella faya* is divided between category 1 (50% of the composition) and categories 2 and 3 (the other 50% of the composition). Finally, the lichen composition of *Erica canariensis* has a bimodal structure, with lichens typical of no eutrophication places (category 1) and species typical of “*rather high eutrophication*” sites (category 4) (Figure 5d). With regard to poleotolerance, *Laurus novocanariensis* composition is equally distributed among categories 0 (“*species, which exclusively occur on old trees in ancient, undisturbed forests*”), 1 (“*occurring in natural or semi-natural habitats*”), and 2 (“*in moderately disturbed areas*”). The lichen composition of *Ilex canariensis* is typical of poleotolerance categories 1 and 2, followed by category 0. The epiphytic lichens of *Morella faya* are linked to category 1, and, to a lesser extent, to category 0. Finally, the lichen composition of *Erica canariensis* has a bimodal structure, with maxima in categories 1 and 3 (“*species occurring in heavily disturbed areas*”) (Figure 5e).

### 3.3. Species Composition

ANOSIM revealed significant differences among four phorophyte combinations (*Laurus novocanariensis* vs. *Erica canariensis*, *Laurus novocanariensis* vs. *Morella faya*, *Erica canariensis* vs. *Ilex canariensis*, and *Morella faya* vs. *Ilex canariensis*), with good separation between *Erica canariensis* and *Ilex canariensis* (R statistic > 0.5; *p* = 6 × 10^−4^), according to Ref [61]. This analysis revealed no significant differences between two combinations of phorophytes (*Laurus novocanariensis* vs. *Ilex canariensis* and *Erica canariensis* vs. *Morella faya*) (Table 4).

The percentages of dissimilarity obtained through the SIMPER analysis were high in all cases, reaching 85%, although the highest percentage values were obtained among the comparisons of differentiated groups (92–98%) and the lowest among *Erica canariensis* vs. *Morella faya* (87%) and *Ilex canariensis* vs. *Laurus novocanariensis* (88%) (Table 4).

The SIMPER analysis revealed discriminant species. Not all species contributed equally to the differences between the phorophytes. A list of characteristic species, with percentages of contribution in brackets, of each phorophyte studied is shown in Table 5. 

Some discriminant species (discriminating species of phorophytes according to the SIMPER analysis; Table 5) are common among phorophytes, this number being higher between *Laurus novocanariensis* and *Ilex canariensis* (four common discriminant species: *Bacidia absistens*, *Lobaria macaronesica*, *Ricasolia virens*, and *Phlyctis agelaea*), between *Erica canariensis* and *Morella faya* (two species: *Chrysothrix candelaris* and *Platismatia glauca*), and between *Ilex canariensis* and *Morella faya* (only *Thelotrema lepadinum*). On the other hand, two discriminant species are common to *Laurus novocanariensis*, *Erica canariensis,* and *Morella faya*: *Parmotrema perlatum* and *Leucodermia leucomela*. No discriminant species is common to all the studied phorophytes.

**Figure 6:**
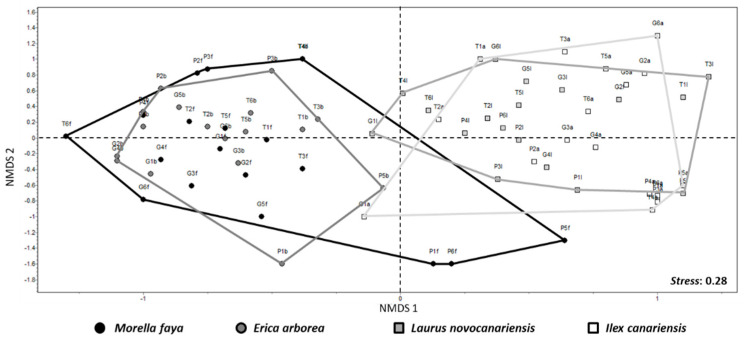
NMDS plot based on abundances matrix and Jaccard distances. Each point represents the sum of lichen abundance in the two trees sampled of each different phorophyte in each plot. *Stress*: 0.28.

NMDS ordination resulted in a two-dimensional pattern with a stress value of 0.28 and revealed a trend in lichen species composition among the studied phorophytes. *Laurus novocanariensis* and *Ilex canariensis* appear on the right side of the plot, whereas *Morella faya* and *Erica canariensis* appear on the left side of the plot, primarily. However, three samples of *Morella faya*, corresponding to plots 7, 11, and 12, and one sample of *Erica canariensis* (plot 11), all from La Palma Island, are on the right side of the NMDS plot. Finally, two samples of *Laurus novocanariensis*, one of them from Tenerife (plot 6) and another from La Gomera (plot 13), are on the left of the NMDS plot (Figure 6). 

## 4. Discussion

### 4.1. Lichen Diversity

Most of the 10 most frequent monteverde species have a wide European distribution (e.g., *Ricasolia virens* and *Bacidia absistens*; Table 6), while others have a much wider global distribution (e.g., *Parmotrema perlatum* and *Heteroderma leucomela*). The endemic element (restricted to the Macaronesian region) is not very numerous, but it is represented by species such as *Lobaria macaronesica*) [62,63,64,65,66]. Some of the most frequently observed species have already been reported as characteristic species of the Canarian Monteverde [19,67,68,69].

The species richness found in the Canarian Monteverde (165 species in total) varies slightly among the phorophytes, with values of 18.3/m^2^ in *Erica* and *Morella*, 20.2/m^2^ in *Laurus*, and 24.7/m^2^ in *Ilex*. Some authors have described that trees with different bark characteristics have different epiphyte species. For this reason, a forest environment in which phorophytes with different barks coexist will accumulate a higher diversity of epiphytes than monospecific forest formations [48,70,71]. 

Although there are no previous studies in the Canarian Monteverde that allow us to compare our data with those studies, we were able to do so with the richness of epiphytic macrolichens identified in a study of the Madeiran monteverde, with a value of 1.4 epiphytic macrolichens/m^2^ [72]. This value is more than 4 times lower than ours if we consider only the macrolichens (5.8 macrolichens/m^2^, 83 species of the 165 observed). It is very relevant to highlight the importance of the contribution of microlichens to the total specific richness.

Studying bryophytes on different phorophytes (*Laurus novocanariensis*, *Morella faya,* and *Erica canariensis*) in Madeira [35], it was found that *Laurus* was the species tree that hosted the most epiphytic species, which agrees with our work in this sense. On the other hand, Ref [73] estimates a value of 5.3 epiphytic bryophytes/m^2^ for *Laurus* in the Monteverde of Tenerife. This value is much lower than the lichen diversity that we observed on this same phorophyte. This same pattern was observed by Ref [74] in the monteverde of the Garajonay National Park (La Gomera island), where lichen diversity (318 species, 52% of them epiphytes) exceeded bryological diversity (199 species, 36.2% of them epiphytes). Although exceptions exist [75], similar behavior has been observed in other forest environments worldwide [76,77,78].

Considering the high frequency of taxa with common traits (biotype, photobiont, etc.), as well as the diversity of the genera and families present in the four phorophytes studied, there seems to be a disjunction in the Canarian Monteverde between the lichens present in *Ilex* and *Laurus* and those that grow in *Morella* and *Erica*, as revealed by the NMDS analysis (Figure 6). Our results are in accordance with the patterns found for bryophytes in previous studies in the Monteverde of Macaronesia, both on the island of Tenerife [32,33] and Madeira [35].

### 4.2. Functional Traits

Functional traits have been used as bioindicators of forest attributes of interest for forest characterization and management [79,80,81]. However, some authors point out that categorizing lichens according to their morphological traits rather than the species, genera, or families to which they belong could lead to erroneous conclusions [82]. For this reason, we will take into account both the functional traits of lichens and their identity.

The study areas were established in homogeneous (meso)climatic zones. Thus, it is expected that the differences found between the two groups of phorophytes described (*Morella* + *Erica* and *Laurus* + *Ilex*) are due to intrinsic characteristics, such as: tree diameter and age, presence of latex and other epiphytes, chemical composition, pH, stability, roughness, thickness and water-holding capacity of the bark, number and size of lenticels, and cortical runoff [27,83,84,85,86,87,88,89,90,91,92,93,94]. Thus, we can define *Morella* and *Erica* as two trees with acid pH, low stemflow funneling ratio values, small leaf area [95,96], and with a thick, rough bark; and *Ilex* and *Laurus* as two trees with neutral pH, high stemflow funneling ratio values, larger leaves [95,96], and smooth and thin bark. None of these trees have a flaking bark or latex.

In terms of the most frequent and diverse taxa found in the studied phorophytes, we observed that crustaceous lichens are very common in *Ilex*. *Erica* is the only phorophyte in which the foliaceous biotype predominates over any other. Crustaceous species richness has been negatively related to tree bark roughness by some authors [30,31]. However, other studies have shown that different crustaceous lichens show different responses to this phorophyte feature [71]. As observed by Refs [30,31], roughness could explain the greater diversity found in *Ilex* (especially) and *Laurus*, since both trees have a smooth bark compared to *Morella* and *Erica*, which have a rough bark. On the other hand, foliaceous species with large thalli, mainly from the *Parmeliaceae* family, show a high biodiversity in *Erica* and *Morella*, where they are more frequent, as well as in *Laurus*. The dichotomy between *Erica* + *Morella* and *Laurus* + *Ilex*, previously mentioned, is further emphasized when studying the dimorphic biotype, represented only by the genus *Cladonia.* It is found exclusively on *Erica* and *Morella*. *Cladonia* has generally been associated with acid substrates, where it predominates [97,98]. This could explain the presence of *Cladonia* exclusively in *Erica* (pH = 5.2 ± 0.7) and *Morella* (pH = 4.5 ± 1.2) and its absence in *Ilex* (pH = 6.4 ± 0.4) and *Laurus* (pH = 6.3 ± 0.6) [95], although variations of this pattern may exist [99]. The *Cladonia* species identified are mostly found in heathlands and acid substrates, both in the monteverde and in other forest environments [69,100,101,102]. In *Erica* and *Morella*, there is also a high frequency of *Chrysothrix candelaris*, a leprose lichen that has been related to acid substrates, rough bark, and environments with high luminosity, both in the Monteverde and in other forest environments [27,69]. However, it has been described as a photophobic species, frequent in acidic and rough crusts [103], which does not tolerate eutrophication [50] and has been observed in well-preserved environments of the Canarian Monteverde [101].

When studying the most frequent species, as well as the most diverse genera and families, we only found cyanolichens (*Sticta canariensis* cyanomorphotype, *Crocodia aurata*, *Sticta*, and *Leptogium*) and tripartite lichens (with green algae as the main photobiont and cyanobacteria as the secondary photobiont: *Ricasolia virens* and *Lobariaceae*) in *Ilex* and *Laurus*. However, no cyanolichens were found among the most frequent species in *Erica* or *Morella*. No representative of this group was found in *Morella*. Although there seem to be exceptions [104], it is generally accepted that cyanolichens need liquid water to photosynthesize [105]. Stemflow funneling is defined by incident rainfall, cortical runoff volume, and diameter at breast height (DBH), but it is also influenced by the roughness of the trunk and branches, as well as by the angle they form with respect to the horizontal. The stemflow funneling ratio data obtained by Ref. [95] for the different trees (with values of 42.5 for *Ilex*, 28.5 for *Laurus*, 22.8 for *Erica,* and 12.7 for *Morella*) support our results. The higher richness of lichens with *Trentepohliaceae* on *Ilex* compared to the rest of the studied phorophytes could be explained by the high number of crustaceous species that develop on this tree. All lichens with this photobiont have this biotype. Finally, the predominance of lichens with chlorococoid green algae found in the monteverde is simply a consequence of their higher abundance in nature [17].

Regarding reproductive strategies, sexual reproduction predominates in the monteverde, followed by multiplication by soredia, isidia, a conjunction of both (soredia and isidia), and fragmentation of the thallus. This pattern has also been described in other forest environments [48,106,107]. However, a more detailed analysis reveals, again, a different pattern among the studied phorophytes. The occurrence of lichens with sexual reproduction is particularly significant in *Ilex* and *Laurus*, while in *Morella* and *Erica*, sexually reproducing species and those that multiply asexually (usually by soredia) co-dominate. Considering that the trees in a given study plot are within a few meters of each other, the lower abundance of sorediate lichens on *Ilex* and *Laurus* compared to *Morella* and *Erica* cannot be explained solely based on the higher difficulty of dispersal of these propagules compared to spores. It has been shown that soredia can reach more than 200 m [108]. Therefore, there must be some feature of the phorophytes that explains this finding. A hypothesis could be that it is related to the bark roughness. Ref. [109] concluded that bark microtopography significantly influenced the establishment and survival of lichens dispersed by soralia; survival was poor on smooth bark compared to survival on rough bark. The smaller size of the spore compared to soredia could explain why species with soralia are less frequent on trees with a smooth bark, where the attachment is more complex compared to that on a rough bark. The same reasoning has been used in the case of bryophyte and fern spores to explain the higher probability of adhesion to a rough surface vs. a smooth surface [110,111,112].

The presence and abundance of some epiphytic lichens in forests are related both to the characteristics of the forest itself and to the ecological requirements of the lichen species [83]. Thus, if we know the ecological requirements of the species inhabiting a forest, we can obtain information about the forest indirectly.

Epiphytic lichens are influenced by macro-, meso-, and microclimatic factors, as well as by the physical and chemical features of the substrate where they grow. Because of these requirements, several authors have used lichens as bioindicators of both polluted and well-preserved environments [113,114]. Most authors adopt the proposal of Ref. [50], using their classification in various regions of Mediterranean influence [48,51,115,116,117]. However, we are not aware of previous studies that have used this methodology to study the specificity of epiphytic lichens and their phorophytes.

Due to the architecture and structure of the studied tree species, the morphology and size of leaves, the characteristics and nature of the bark affecting the water-holding capacity, runoff, etc., a differential response of lichens to solar radiation, aridity, and even pH would be expected. Regarding pH, it should be noted that lichens found exclusively on *Laurus* and *Ilex* have an affinity for subacid to subneutral substrates, and these trees are the only ones with lichens species with an affinity for slightly basic substrates. However, the epiphytic lichens growing only on *Morella* and *Erica* are associated with acidic and subacid to subneutral substrates, with no exclusive lichen species having an affinity for (slightly) basic substrates. This is consistent with the previous comments on the pH characteristics of the four phorophytes and the occurrence of particular groups of lichens associated with this character. 

In terms of the response of lichens to solar irradiation, we expected to obtain a certain homogeneity due to the similar characteristics of the canopy of the different localities and the fact that all are evergreen and broadleaf trees, with the exception of *Erica*, which has substantially different leaves. In the case of *Ilex*, *Laurus,* and *Morella*, we expected to obtain a significant proportion of poorly light-tolerant lichens, typical of shady environments, such as evergreen forests like the Canarian Monteverde (category 1 sensu Ref. [50]). We did not find a common or distinctive pattern in the trees studied, nor was our hypothesis regarding ombrophilous lichens fulfilled (Figure 5b). However, we were able to detect certain trends. Lichens growing on *Laurus* and *Ilex* seem to have a certain affinity for environments with a lot of diffuse light but little direct solar irradiation, while the response to light in the epiphytes of *Morella* and *Erica* is completely opposite and different from *Laurus* and *Ilex*. In *Morella*, sciaphilous species dominate, with almost 50% of its epiphytes in category 2, while *Erica* is dominated by species with very high direct solar irradiation requirements, with more than half of its species in category 5. Both *Morella* and *Erica* are heliophilous and primocolonizing species of the Canarian Monteverde [118]. However, *Erica* does not usually take part in mature Monteverde communities, while *Morella* remains, even being part of the mature (climax) phase of the forest [119].

On the other hand, the leaves of *Erica* are by far the smallest and narrowest of the four trees analyzed [95,96]. Therefore, it would be expected that higher solar radiation penetrates under its canopy, and consequently, more lichens with high light requirements would grow on its bark, as is the case here. The leaves of *Morella*, although some authors considered them small compared to those of *Laurus* or *Ilex* [95], are much wider and longer than those of *Erica*. This would explain the higher abundance of lichens related to shady environments with respect to *Erica* but less in relation to *Laurus* or *Ilex*. 

As a response to the aridity, it could be hypothesized that the epiphytic lichens of all monteverde trees studied have ecological indicator values for this indicator, which are typical of hygrophytic environments (low values), as it is a forest linked to high humidity [120]. *Erica* epiphytes tend to tolerate a higher degree of aridity than expected (Figure 5c). This could be explained partly by the solar irradiance data mentioned above and also by the low channeling values of this tree [95]. In the case of *Morella*, although the channeling values are not high [95], the water retention capacity of the bark of this tree, particularly in the larger and corky-looking specimens, could be playing a significant role. This could explain why this phorophyte has the highest percentage of species classified as slightly hygrophytic. Overall, *Laurus* and *Ilex* could be classified as two trees with epiphytic lichens typical of a mesophytic to slightly hygrophytic environment. Considering the four phorophytes analyzed in this study, this forest could be defined as a mesophytic to slightly hygrophytic forest environment.

For the eutrophication and poleotolerance, low values were expected for the four phorophytes analyzed, as it is a primary forest. The values for *Ilex* and *Laurus* are in accordance with those expected, with a high proportion of lichens occurring exclusively on old trees in ancient, undisturbed forests or natural (or semi-natural) habitats. Consequently, they are mostly non-resistant to eutrophication or resistant to a very weak eutrophication (Figure 5d,e). *Morella* also has a unique diversity of epiphytic lichen diversity with low eutrophication and poleotolerance values. The case of *Erica* is particular in this respect, showing the highest values of eutrophication and poleotolerance. These high values are related to the presence of a single species: *Amandinea punctata*. It is responsible not only for the maximum observed in these indicators but also has a great impact on the solar irradiation values. This species has only been found in one plot on *Erica* on the island of La Palma but with an astonishing abundance. It was reported for the first time for the Canary Islands in the Caldera de Taburiente National Park (La Palma) on various substrates [121], but it has also been collected in the monteverde of the Garajonay National Park (La Gomera) on rocky outcrops in open and somewhat degraded areas of *Morella* and *Erica* forest [20], and in El Teide National Park in Tenerife [122]. The diversity of the environments in which this species has been reported shows its wide ecological value and it should not be considered in any case as a bioindicator species of the Canarian Monteverde. As previously stated, we always used the maximum ecological value, and therefore, the analysis may be affected in this case.

### 4.3. Lichen Composition

The low proportion of shared lichens among all the studied phorophytes compared to the diversity found exclusively in each phorophyte, suggests a possible lichen–phorophyte specificity. The specificity of epiphytic lichens and their phorophytes is a controversial issue, and it does not seem that the same patterns occur in all forest ecosystems. Thus, while some authors report some specificity [28,29], others do not see a clear pattern [30,123], and some authors even suggest that the results obtained in this regard in plurispecific forests should be taken with some cautiousness [42,124].

The high species richness shared between *Erica* and *Morella* on the one hand, and between *Laurus* and *Ilex* on the other, suggests a possible affinity of lichens for the trees that comprise these two subsets. In this case, this pattern seems to be consistent with what has been reported by other authors in tropical forests. Several authors have found similar patterns, i.e., affinity of lichen species for groups of phorophytes, and have explained them based on the physical and chemical features of the trees and their bark (pH, roughness, water retention capacity, tree perimeter, etc.) [30,31,85,125]. It should also be noted that the two aforementioned subgroups (*Laurus novocanariensis* + *Ilex canariensis* and *Morella faya* + *Erica canariensis*) are the same as those observed by Refs. [32,70] when studying epiphytic bryophytes in the monteverde of La Gomera.

According to Ref. [30], the specificity of epiphytes to phorophytes is not beneficial for lichens in forests with a plurispecific tree composition, as it decreases the probability of finding and establishing lichen diaspores with a suitable phorophyte. This can be an issue when the diaspores are asexual propagules, as they are larger and heavier than sexual spores and have lower dispersal facility [126,127,128]. This has implications for the conservation of monteverde, particularly on islands such as Tenerife, where the forest is fragmented [2], and should be considered in ecological restoration actions in the Canarian Monteverde.

## 5. Conclusions

No specificity of Canarian Monteverde lichens concerning the tree species analyzed was observed. However, an affinity toward two well-defined groups of phorophytes was identified: *Erica* + *Morella* and *Laurus* + *Ilex*. This differentiation is clear, considering the specific diversity and the number of lichen species shared by these two groups. These data are also supported by the patterns of functional traits of lichens that comprise these two groups. The differences found may respond to the physico-chemical features of the tree (leaf type and canopy structure, roughness, pH, and thickness of the bark, among others). Further studies would be necessary to elucidate the importance of each of these factors separately. The results presented will be useful to improve the ecological restoration efforts in the Canarian Monteverde.

## Figures and Tables

**Table 1:** Field plots.

Plot	Island	Locality	Universal Transverse Mercator (UTM)
1	Tenerife	Pista Las Hiedras (Anaga)	375110/3157614
2	Tenerife	Erjos (Teno)	321907/3134428
3	Tenerife	Cueva del Guanche (Anaga)	380235/3157573
4	Tenerife	Aguagarcía	362558/3148881
5	Tenerife	Hoya de Ijuana (Anaga)	385900/3159815
6	Tenerife	Las Portelas (Teno)	320500/3135776
7	La Palma	Mirador de Las Mimbreras	223103/3190532
8	La Palma	Don Pedro	216864/3191489
9	La Palma	Laguna de Barlovento	225903/3190442
10	La Palma	Hoyo del Infierno	226243/3168304
11	La Palma	Roque Niquiomo	225640/3166681
12	La Palma	La Pavona	225015/3170197
13	La Gomera	Lomito Ventoso	273628/3116431
14	La Gomera	Cordilleras de Vallehermoso	276756/3115483
15	La Gomera	El Bailadero	283088/3112836
16	La Gomera	El Contadero	279720/3112282
17	La Gomera	Fuensanta	278779/3115994
18	La Gomera	La Gollada Colorada	278663/3114210

**Table 2:** Functional traits: Photobiont, growth form, and reproductive strategy.

Functional Traits	Types
Photobiont	Green algae
	*Trentepohliaceae*
	Cyanobacteria
	Without thallus
Growth form	Leprose/Pulverulent
	Crustose
	Squamulose
	Foliose
	Fruticose
	Cladoniiform
Reproductive strategy	Thallus fragmentation
	Isidia
	Soralia
	Isidia + soralia
	Sexual (spores)
	Conidia

**Table 3:** Functional traits: Ecological requirement. Extracted from Ref. [50].

Functional Traits	Value	Signification
Tolerance to eutrophication	1	Lichens not resistant to eutrophication
	2	Lichens resistant to a very weak eutrophication
	3	Lichen resistant to a weak eutrophication
	4	Lichen occurring in rather eutrophicated situations
	5	Lichens occurring in highly eutrophicated situations
Water requirements	1	Hydro and hygrophytic species, in sites with a very high frequency of fog
	2	Rather hygrophytic species, intermediate between 1 and 3
	3	Mesophytic species
	4	Xerophytic species, but absent from extremely arid stands
	5	Very xerophytic species
Solar irradiation	1	Species growing in very shaded situations
	2	Species growing in shaded situations
	3	Species growing in sites with plenty of diffuse light but scarce direct solar irradiation
	4	Species growing in sun-exposed sites, but avoiding extreme solar irradiation
	5	Species growing in sites with very high direct solar irradiation
Poleotolerance	0	Lichens that occur exclusively on old trees in ancient, undisturbed forests
	1	Lichens mostly occurring in natural or semi-natural habitats
	2	Lichens also occur in moderately disturbed areas (agricultural areas, small settlements, etc.)
	3	Lichens also occur in heavily disturbed areas, incl. large towns
Substrata pH	1	Species, which occur on very acid substrata
	2	Species, which occur on acid substrata
	3	Species, which occur on subacid to subneutral substrata
	4	Species, which occur on slightly basic substrata
	5	Species, which occur on basic substrata

**Table 4:** ANOSIM and SIMPER. ANOSIM values above the diagonal (significant differences shown with *) and SIMPER values on the diagonal and under it (*: *p*-value ≤ 8.3 × 10^−3^).

	*Morella*	*Erica*	*Laurus*	*Ilex*
***Morella***	8.9	0.084	0.35 *	0.38 *
***Erica***	87%	22	0.4 *	0.59 *
***Laurus***	96%	92%	14	0.033
***Ilex***	98%	98%	88%	11

**Table 5:** SIMPER results. Discriminant species of *Ilex canariensis*, *Laurus novocanariensis*, *Erica canariensis,* and *Morella faya*. We showed species with 75% of total contribution to each group. Percentages of contribution per species are shown in brackets.

***Ilex canariensis***	***Laurus novocanariensis***
*Phlyctis agelaea* (14%) *Ricasolia virens* (10%) *Bacidia absistens* (8.4%) *Bacidia herbarum* (8.1%) cf. *Phyllopsora* (6.4%) *Athallia holocarpa* (6.3%) *Lecanora pulicaris* (4.9%) *Lobaria macaronesica* (4.3%) *Sticta canariensis cyanomorph.* (4%) *Thelotrema lepadinum* (3.6%) *Lecidella elaeochroma* (3.6%) *Lecanora rubicunda* (3%)	*Parmotrema perlatum* (22%) *Ricasolia virens* (13%) *Phlyctis agelaea* (11%) *Leucodermia leucomela* (9.3%) *Lobaria macaronesica* (6.8%) *Crocordia aurata* (5.7%) *Bacidia absistens* (3.9%) *Lobaria immixta* (3%)
***Erica canariensis***	***Morella faya***
*Parmotrema perlatum* (32%) *Chrysothrix candelaris* (28%) *Parmotrema reticulatum* (6.1%) *Leucodermia leucomela* (6%) *Platismatia glauca* (5.9%)	*Parmotrema perlatum* (20%) *Thelotrema lepadinum* (17%) *Parmotrema crinitum* (9.3%) *Parmelinopsis horrescens* (8.8%) *Chrysothrix candelaris* (7.4%) *Platismatia glauca* (5.8%) *Lecanora albella* (4.1%) *Leucodermia leucomela* (2.7%)

**Table 6:** Ten most frequent monteverde species and their absolute presences and worldwide distribution. * sensu Ref. [65].

Taxa	Presences	Worldwide Distribution *
*Parmotrema perlatum*	202	Cosmopolitan (except Africa)
*Chrysothrix candelaris*	142	Cosmopolitan
*Leucodermia leucomelos*	117	Cosmopolitan
*Phlyctis agelaea*	84	Holarctic (mainly Europe)
*Bacidia absistens*	73	Holarctic (mainly Europe)
*Ricasolia virens*	73	Holarctic (mainly Europe)
*Platismatia glauca*	62	Cosmopolitan (except Australia)
*Lobaria macaronesica*	56	Macaronesia
*Lepra slesvicensis*	52	Cosmopolitan
*Parmotrema crinitum*	48	Cosmopolitan

## Data Availability

Not applicable.

## Supplementary Materials

The following supporting information can be downloaded at: https://www.mdpi.com/article/10.3390/jof8101031/s1, Table S1: Lichen checklist. Table S2: Ecological indicator values and functional traits of the studied lichens.
