# Supplementary material for: Together Apart: Evaluating Lichen-Phorophyte Specificity in the Canarian Laurel Forest
Source: J Fungi (Basel). 2022 Sep 29;8(10):1031. doi: 10.3390/jof8101031 (PMC9605454; doi:10.3390/jof8101031)
Supplement: Supplementary file 1 [file jof-08-01031-s001.zip › Table S1.pdf]

Table S1. Lichen checklist.

Taxa	TENERIFE				LA PALMA				LA GOMERA			
	Morella	*Erica*	*Laurus*	*Ilex*	*Morella*	*Erica*	*Laurus*	*Ilex*	*Morella*	*Erica*	*Laurus*	*Ilex*
Acrocordia gemmata (Ach.) A. Massal.												+
Alectoria imshaugii Brodo & D. Hawksw.										+		
Amandinea punctata (Hoffm.) Coppins & Scheid.						+						
Anisomeridium polypori (Ellis & Everh.) M.E. Barr								+				
Anisomeridium ranunculosporum (Coppins & P. James) Coppins								+				
Reichlingia anombrophila (Coppins & P. James) Frisch									+			
Arthonia ilicina Taylor												+
Coniocarpon cinnabarinum DC.	+			+								
Arthonia stellaris Kremp.								+				
***Arthonia* sp. 1**	+											
Bacidia absistens (Nyl.) Arnold						+	+	+			+	+
Bacidia arceutina (Ach.) Arnold								+				
Bacidia herbarum (Stizenb.) Arnold							+	+				
Bacidia laurocerasi (Delise *ex* Duby) Zahlbr.		+					+	+				
Bacidia rosella (Pers.) De Not.								+				
Bacidia rubella (Hoffm.) A. Massal.							+	+				
Bibbya vermifera (Nyl.) Kistenich, Timdal, Bendiksby & S. Ekman							+					
Bryoria chalybeiformis (L.) Brodo & D. Hawksw.					+							
Bryoria furcellata (Fr.) Brodo & D. Hawksw.										+		
Bryoria fuscescens (Gyeln.) Brodo & D. Hawksw.						+				+		
Buellia disciformis (Fr.) Mudd			+	+		+	+					
Buellia leptoclinoides (Nyl.) J. Steiner	+											
Byssoloma marginatum (Arnold) Sérus.						+						
Byssoloma subdiscordans (Nyl.) P. James					+	+	+	+			+	
Calicium glaucellum Ach.						+						
Blastenia ferruginea (Huds.) A. Massal.				+			+	+				
Athallia holocarpa (Hoffm.) Arup, Frödén & Søchting			+	+			+	+				

Taxa	TENERIFE				LA PALMA				LA GOMERA			
	Morella	*Erica*	*Laurus*	*Ilex*	*Morella*	*Erica*	*Laurus*	*Ilex*	*Morella*	*Erica*	*Laurus*	*Ilex*
Chrysothrix candelaris (L.) J.R. Laundon	+	+			+	+	+		+	+		
Cladonia carneola (Fr.) Fr.		+				+						
Cladonia coniocraea (Flörke) Spreng.	+	+				+						
Cladonia fimbriata (L.) Fr.										+		
Cladonia floerkeana (Fr.) Flörke		+										
Cladonia macilenta Hoffm.	+											
Cladonia merochlorophaea Asahina									+	+		
Cladonia parasitica (Hoffm.) Hoffm.	+	+										
Cladonia squamosa (Scop.) Hoffm.	+	+										
Cladonia subulata (L.) Weber *ex* F.H. Wigg.		+										
Coenogonium luteum (Dicks.) Kalb & Lücking	+	+					+		+		+	
Coenogonium pineti (Ach.) Lücking & Lumbsch							+				+	
Collema subnigrescens Degel.				+								
Pectenia atlantica (Degel.) P.M. Jørg., L. Lindblom, Wedin & S. Ekman			+	+								
Pectenia ligulata (P.M. Jørg. & P. James) P.M. Jørg., L. Lindblom, Wedin & S. Ekman			+	+								
Pectenia plumbea (Lightf.) P.M. Jørg., L. Lindblom, Wedin & S. Ekman			+	+			+	+				
Fuscopannaria mediterranea (Tav.) P.M. Jørg.										+		+
Graphis scripta (L.) Ach.	+											
Leucodermia boryi (Fée) Kalb									+	+	+	+
Polyblastidium japonicum (M. Satô) Kalb										+	+	
Leucodermia leucomelos (L.) Kalb	+	+	+	+			+	+	+	+	+	+
Heterodermia obscurata (Nyl.) Trevis.	+	+							+			
Hypogymnia physodes (L.) Nyl.		+							+			
Hypogymnia tubulosa (Schaer.) Hav.		+										
Hypotrachyna endochlora (Leight.) Hale	+	+							+	+		
Hypotrachyna laevigata (Sm.) Hale	+	+			+				+	+		
Hypotrachyna revoluta (Flörke) Hale		+			+	+			+	+		
Hypotrachyna rockii (Zahlbr.) Hale	+											
Hypotrachyna sinuosa (Sm.) Hale					+							
Hypotrachyna taylorensis (M.E. Mitch.) Hale		+										
Lecanactis abietina (Ach.) Körb.									+			
Lecanora albella (Pers.) Ach.					+	+	+					

Taxa	TENERIFE				LA PALMA				LA GOMERA			
	Morella	*Erica*	*Laurus*	*Ilex*	*Morella*	*Erica*	*Laurus*	*Ilex*	*Morella*	*Erica*	*Laurus*	*Ilex*
Lecanora allophana (Ach.) Nyl.			+									
Lecanora argentata (Ach.) Röhl.			+	+			+	+				
Lecanora chlarotera Nyl.			+	+			+	+				
Lecanora gangaleoides Nyl.				+								
Lecanora hybocarpa (Tuck.) Brodo			+	+								
Lecanora pulicaris (Pers.) Ach.			+	+	+		+	+				+
Lecanora rubicunda Bagl.			+	+			+	+				
Lecanora rugosella Zahlbr.							+	+				
Lecanora symmicta (Ach.) Ach.				+								
Lecidella elaeochroma (Ach.) M. Choisy					+		+	+				
Scytinium aragonii (Otálora) Otálora, P.M. Jørg. & Wedin			+									
Leptogium brebissonii Mont.											+	+
Leptogium cochleatum (Dicks.) P.M. Jørg. & P. James			+				+				+	+
Leptogium coralloideum (Meyen & Flot.) Vain.							+				+	
Leptogium cyanescens (Ach.) Körb.			+	+							+	+
Leptogium* aff. *cyanescens											+	
Scytinium tenuissimum (Hoffm.) Otálora, P.M. Jørg. & Wedin			+									
Leptogium teretiusculum Wallr. *ex* Arnold							+				+	+
Lobaria immixta Vain.			+	+			+	+			+	+
Lobaria macaronesica C. Cornejo & Scheid.		+	+	+			+				+	+
Ricasolia virens (With.) H.H. Blom & Tønsberg			+	+			+				+	+
Micarea alabastrites (Nyl.) Coppins	+	+			+	+			+	+		
Micarea pycnidiophora Coppins & P. James					+	+			+	+		
Micarea stipitata Coppins & P. James					+	+			+	+		
Micarea synotheoides (Nyl.) Coppins										+		
Megalaria grossa (Pers. *ex* Nyl.) Hafellner								+				
Mycoporum lacteum (Ach.) R.C. Harris					+							
Nephroma laevigatum Ach.		+	+									
Normandina pulchella (Borrer) Nyl.			+				+	+		+	+	+
Ochrolechia balcanica Verseghy				+								
Ochrolechia pallescens (L.) A. Massal.			+	+								
Ochrolechia szatalaensis Verseghy				+								
Alyxoria culmigena (Lib.) Ertz	+	+		+				+				

Taxa	TENERIFE				LA PALMA				LA GOMERA			
	Morella	*Erica*	*Laurus*	*Ilex*	*Morella*	*Erica*	*Laurus*	*Ilex*	*Morella*	*Erica*	*Laurus*	*Ilex*
Alyxoria ochrocincta (Werner) Ertz								+				
Alyxoria varia (Pers.) Ertz & Tehler								+				
Pannaria rubiginosa (Ach.) Delise			+								+	+
Parmelia saxatilis (L.) Ach.	+	+				+				+		
Parmelia sulcata Taylor					+							
Parmelinopsis horrescens (Taylor) Elix & Hale	+								+	+		
Hypotrachyna minarum (Vain.) Krog & Swinscow	+				+	+						
Parmotrema arnoldii (Du Rietz) Hale									+	+		
Parmotrema crinitum (Ach.) M. Choisy	+	+							+	+	+	
Parmotrema perlatum (Huds.) M. Choisy	+	+	+	+	+	+	+	+	+	+	+	
Parmotrema reticulatum (Taylor) M. Choisy	+	+		+	+	+				+	+	
Parmotrema robustum (Degel.) Hale	+	+										
Lepra albescens (Huds.) Hafellner	+	+	+	+								
Lepra amara (Ach.) Hafellner	+	+	+	+		+	+		+		+	+
Pertusaria coccodes (Ach.) Nyl.	+											
Pertusaria dispar J. Steiner				+								
Pertusaria hymenea (Ach.) Schaer.								+				
Pertusaria leioplaca (Ach.) DC.							+	+				
Lepra multipuncta (Turner) Hafellner				+								
Lepra ophthalmiza (Nyl.) Hafellner	+		+	+					+	+		+
Lepra slesvicensis (Erichsen) Hafellner	+	+	+	+					+	+		
Varicellaria velata (Turner) I. Schmitt & Lumbsch	+			+								
Pertusaria werneriana Boqueras				+								
Phlyctis agelaea (Ach.) Flot.			+	+			+	+			+	+
Phlyctis argena (Ach.) Flot.			+	+								
***Phyllopsora* cf.**									+		+	+
Piccolia ochrophora (Nyl.) Hafellner							+					
Pyrenula dermatodes (Borrer) Schaer.												+
Platismatia glauca (L.) W.L. Culb. & C.F. Culb.	+	+				+			+	+		
Pseudosagedia aenea (Körb.) Hafellner & Kalb				+								
Porina atlantica (Erichsen) P.M. Jørg.											+	
Porina effilata M. Brand & Sérus.												+
Crocodia aurata (Ach.) Link		+	+	+			+	+			+	+
Pseudocyphellaria crocata (L.) Vain.											+	

Taxa	TENERIFE				LA PALMA				LA GOMERA			
	Morella	*Erica*	*Laurus*	*Ilex*	*Morella*	*Erica*	*Laurus*	*Ilex*	*Morella*	*Erica*	*Laurus*	*Ilex*
Pseudocyphellaria intricata (Delise) Vain.											+	+
Pyrenula occidentalis (R.C. Harris) R.C. Harris	+			+								
Pyrenula pseudobufonia (Rehm) R.C. Harris				+								
Ramalina chondrina J. Steiner							+	+				
Ramalina farinacea (L.) Ach.			+	+			+					
Ramalina fastigiata (Pers.) Ach.						+	+					
Ramalina peruviana Ach.							+					
Ramalina pusilla Le Prévost *ex* Duby							+					
Ramalina subgeniculata Nyl.							+	+				
Rinodina capensis Hampe								+				
Rinodina septentrionalis Malme								+				
Sphaerophorus globosus (Huds.) Vain.									+	+		
Sphinctrina leucopoda Nyl.								+				
Stenocybe septata (Leight.) A. Massal.	+											
Sticta canariensis (Bory) Bory *ex* Delise (chloromorph.)			+	+								
Sticta canariensis (Bory) Bory *ex* Delise (cyanomorph)				+			+				+	+
Sticta fuliginoides Magain & Sérus.											+	+
Sticta ciliata Taylor										+	+	+
Sticta fuliginosa (Dicks.) Ach.											+	+
Sticta limbata (Sm.) Ach.				+		+	+				+	+
Strigula tagananae (Harm.) R.C. Harris				+								
Syncesia myrticola (Fée) Tehler	+											
Teloschistes flavicans (Sw.) Norman	+	+	+	+			+					
Tephromela atra (Huds.) Hafellner							+					
Thelotrema laurisilvae Lücking & Breuss												+
Thelotrema lepadinum (Ach.) Ach.	+	+		+					+		+	+
Thelotrema macrosporum P.M. Jørg. & P. James				+								
Nephromopsis chlorophylla (Willd.) Divakar, A. Crespo & Lumbsch					+				+	+		
Usnea barbata (L.) F.H. Wigg.	+											
Usnea chaetophora Stirt.	+											
Usnea cornuta Körb.	+			+		+				+		
Usnea diplotypus Vain.	+					+				+		
Usnea flammea Stirt.	+	+			+	+						

Taxa	TENERIFE				LA PALMA				LA GOMERA			
	Morella	***Erica***	***Laurus***	***Ilex***	***Morella***	***Erica***	***Laurus***	***Ilex***	***Morella***	***Erica***	***Laurus***	***Ilex***
Usnea schadenbergiana Göpp. & Stein	+	+										
Usnea subflammea P. Clerc	+											
Usnea subfloridana Stirt.	+											
Usnea subscabrosa Nyl. *ex* Motyka	+	+		+		+			+	+		
Vahliella saubinetii (Mont.) P.M. Jørg.											+	
***Vezdaea* sp. 1**					+							
